# The Impact of Harvesting Mechanization on Oolong Tea Quality

**DOI:** 10.3390/plants13040552

**Published:** 2024-02-18

**Authors:** Junling Zhou, Shuilian Gao, Zhenghua Du, Tongda Xu, Chao Zheng, Ying Liu

**Affiliations:** 1 College of Horticulture, Haixia Institute of Science and Technology, Fujian Agriculture and Forestry University, Fuzhou 350007, China; junling-zhou@fafu.edu.cn (J.Z.); gaoshuilian@126.com (S.G.); zhenghuadu@fafu.edu.cn (Z.D.); 2 Anxi College of Tea Science, Fujian Agriculture and Forestry University, Fuzhou 350007, China

**Keywords:** UPLS-QToF-MS, oolong tea, harvesting mechanization, sensory, untargeted metabolomics, chemometric, quality control

## Abstract

Mechanization is the inevitable future of tea harvesting, but its impact on tea chemistry and quality remains uncertain. Our study examines untargeted metabolomic data from 185 oolong tea products (Tieguanyin) made from leaves harvested by hand or machine based on UPLC-QToF-MS analysis. The data revealed a minimum 50% loss for over half of the chemicals in the machine-harvested group, including catechins, theaflavin, gallic acid, chlorogenic acid, and kaempferol-3-gluocside. Integrating sensory evaluation, OPLS-DA identified the six most important metabolites as significant contributors to sensory decline caused by harvesting mechanization. Furthermore, our research validates the possibility of using DD-SIMCA modelling with untargeted metabolomic data for distinguishing handpicked from machine-harvested tea products. The model was able to achieve 93% accuracy. This study provides crucial insights into the chemical and sensory shifts during mechanization, along with tools to manage and monitor these changes.

## 1. Introduction

Tea (*Camellia sinensis* (L.) O. Kuntze) is the most consumed beverage in the world besides water. Its popularity is based on the pleasant relaxing feeling during consumption and the widely accepted health benefits associated with tea [1]. Both reasons are associated with the complex phytochemicals in tea infusions. Flavan-3-ols, theanine, caffeine, and polysaccharides have been reported with hyperglycemic, hypolipidemic, anti-inflammatory, and preventative effects on tumorigenesis activities [2]. The pleasant smell is mainly attributed to the volatile organic compounds in the tea leaves, the oxidation of fatty acids, and carotenoids [3]. The unique taste is a result of secondary metabolites as well. For example, flavanol-*O*-glycosides, tannins, and galloylated catechins contribute to the astringent taste, and L-theanine, succinic acid, gallic acid, and theogallin contribute to the umami taste [1].

The phytochemistry of tea is shaped by many factors. Tea research often focuses on how pre-harvesting and/or post-harvesting treatment can impact tea chemistry but tends to neglect the process of actual harvesting. Pre-harvest studies investigate the impact of environmental factors, such as climate [4] and altitude [5], on the chemistry of fresh leaves, while post-harvesting research studies how each processing step, such as the degree of fermentation [6] and temperature of roasting [7], alters the chemical transformation from the fresh leaves to final products. All these factors are without doubt important in shaping tea chemistry. However, the harvesting process itself is also important since the inappropriate collection of the leaf materials can single-handedly diminish the whole product [8,9,10,11]. 

Traditionally, tea harvesting was conducted by the meticulous removal of buds and two to four leaves from the tips of tea bushes. This manual approach focuses on picking only fresh young leaves which ensures the highest tea quality. But it demands over 70% of the workforce in the tea industry, contributing to more than 35% of production costs [12,13]. During the 1900s, Japan developed the first mechanized plucking machines for tea which marked the beginning of the global adoption of mechanized tea harvesting. Subsequently, countries such as Russia, England, France, India, Australia, and Argentina embraced the mechanization of tea harvesting [14,15]. China is the world’s largest tea producer. The country produces around 45% of the global total, and is one of the three major tea exporters [16]. China started the mechanization of tea harvesting in the 1970s and is currently in a semi-mechanized state [14]. 

While it is widely acknowledged that handpicked tea is associated with higher quality, there is limited research on the chemical profile differences between handpicked and machine-harvested tea. The only study was conducted in 1998 by Ravichandran and Parthiban [17]. The study observed a reduction in total catechins and polyphenols when comparing handpicked and machine-harvested leaves. Both catechins and polyphenols, such as theaflavins and thearubigins measured in the study, contribute significantly to the color, strength, and mouth-feel of tea liquor [18]. However, given the advancements in analytical chemistry and harvesting mechanics since 1998, it is necessary to construct a more comprehensive understanding of the metabolic changes resulting from mechanization.

Untargeted metabolomics, an unbiased approach, has gained widespread adoption for unveiling the chemistry of tea [19]. Utilizing analytical tools such as UPLC/MS (ultra-performance liquid chromatography/mass spectrometry), GC-ToF/MS (Gas chromatography/mass spectrometry), and NMR (nuclear magnetic resonance) allows the generation of thousands of chemical data, providing a comprehensive overview of tea metabolites [20]. These data sets are further analyzed using chemometrics. Chemometrics refers to the application of mathematical methods like PCA (principal component analysis), OPLS-DA (orthogonal partial least squares-discriminant analysis), and PLS-DA (partial least squares discriminant analysis) to solve chemical problems [21]. Integration of untargeted metabolomic data with chemometrics empowers researchers to extract maximum information, enabling the characterization of tea chemistry and identification of potential markers in diverse tea research studies [22,23,24,25,26,27].

Oolong tea is one of the six types of tea that share characteristics with both green and black tea. The tea is famous for its elegant fruity taste and floral fragrance. The chemical compounds clarify and intensify during the semi-fermentation process which involves gradual dehydration and moderate bruising [28]. Anxi Tieguanyin, a renowned oolong tea, contributes to over 40% of China’s total oolong tea production. The cultivar serves as a valuable example case for understanding the impact of mechanization in the tea industry. 

Therefore, this investigation employed UPLC-QToF-MS (ultra-performance liquid chromatography-quadrupole time-of-flight mass spectrometry), along with chemometric modelling to understand the phytochemical changes caused by harvesting mechanization. The data presented in this study provided the scientific base for the transition to harvesting mechanization. It offers potential quality control measures to oversee and manage the impact mechanization imposes on tea quality. 

We aim to understand the nature and extent of metabolite changes associated with mechanization. A total of 185 Tieguanyin oolong tea products manufactured from leaves harvested both manually and mechanically at the same production site were collected, and a complete phytochemical profile was collected using untargeted metabolomics. To understand how these chemical changes may alter the quality of the tea, all samples underwent a comprehensive sensory assessment to establish a correlation between chemical shifts and sensory grading and identify the most severe potential chemical markers. We further showcase the viability of utilizing metabolite data and chemometric modelling to distinguish between handpicked and machine-harvested oolong tea, suggesting a new direction for quality control. 

## 2. Results and Discussion

### 2.1. Untargeted Metabolomics of Handpicked and Machine-Harvested Tea Products

**Figure 1:**
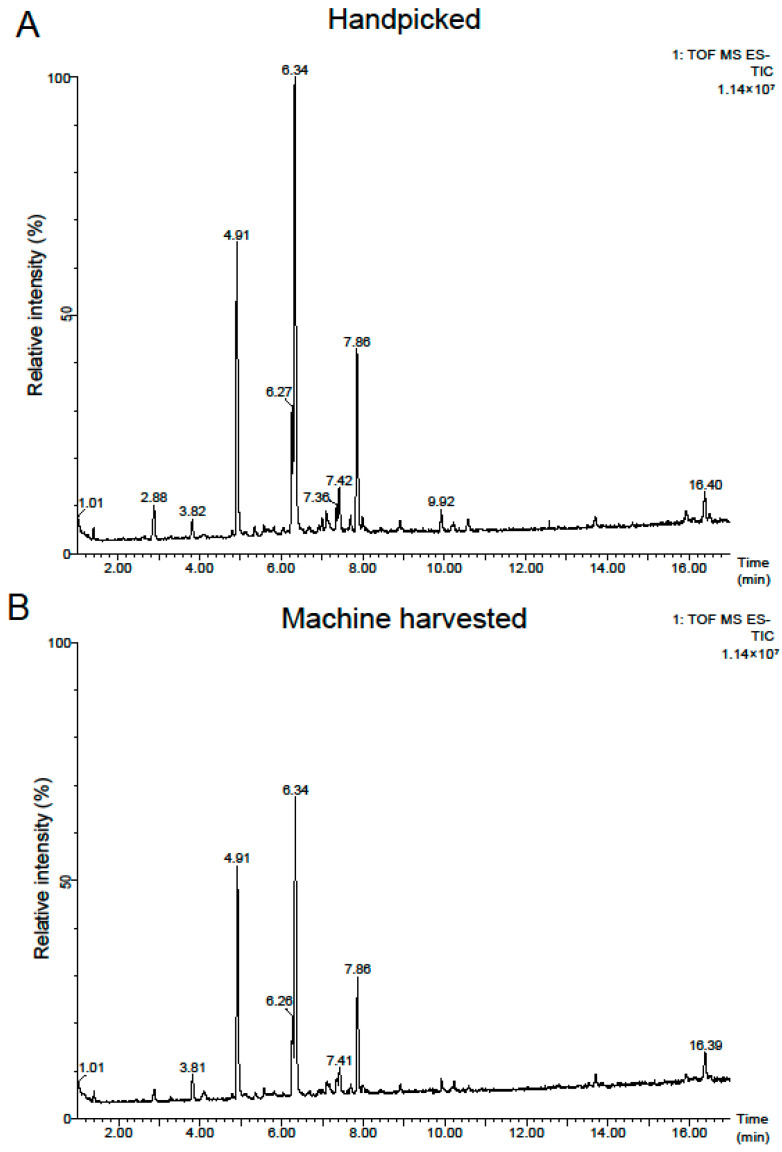
TICs of Tieguanyin tea products made from handpicked (**A**) and machine-harvested leaves (**B**).

Tea products (*Camellia sinensis* var. *sinensis* cv. ‘Tieguanyin’) made from handpicked and machine-harvested leaves were obtained during the autumn of 2022. All the tea plants were from the same production site. The metabolomic profiles of the final tea products were assessed in order to explore the impact of harvesting method on the metabolic profiles of these teas. Figure 1 showcases a chromatogram illustrating the total ion current (TIC) of representative tea samples, analyzed using a UPLC-QToF-MS in ESI (electrospray ionization) negative mode. A total of 1967 distinct features were identified with 182 metabolites successfully annotated. The detailed annotation information of the metabolites is presented in Table S1. The majority (53.8%) of these metabolites belong to the flavonoid category, encompassing flavonoid glycosides, flavans, bioflavonoids, and polyflavonoids, followed by prenol lipids (13.7%) which are triterpene saponins (Figure S1).

**Figure 2:**
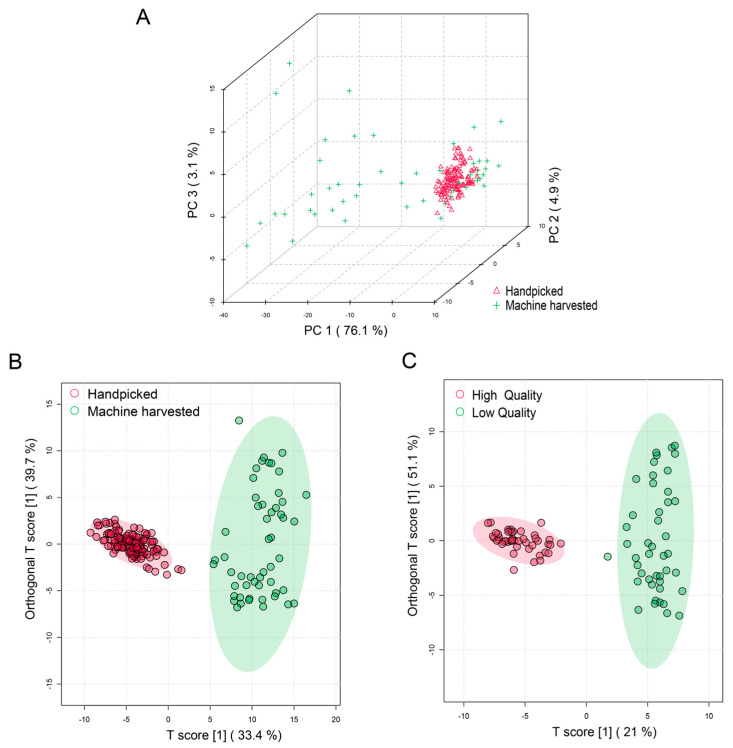
(**A**) PCA score plot of Tieguanyin tea products made from handpicked and machine-harvested leaves; (**B**) OPLS-DA score plot of Tieguanyin tea products made from handpicked and machine-harvested leaves; (**C**) OPLS-DA score plot of high- and low-grade Tieguanyin tea products.

An initial unsupervised principal component analysis (PCA) model (Figure 2A) showed that samples from the machine-harvested tea groups are scattered across the region. The handpicked group had a high conformity despite having a much larger sample size. Almost all the annotated metabolites were higher in the handpicked group than the machine-harvested group. Using OPLS-DA (orthogonal partial least squares-discriminant analysis), we discovered 85 key differential metabolites with VIP > 1.0, *p* < 0.05 (Figure 2B). Most of them (64 out of 84) experienced at least 1.5-fold changes. About 17 metabolites experienced over 2-fold of changes. The most significant changes happened with oleiferasaponin C5, unknown 6, echinocystic acid 3-*O*-[beta-*D*-galactopyranosyl (1->2)]-[beta-*D*-glucopyranosyl (1->2)-beta-*D*-galactopyranosyl (1->3)]-beta-*D*-glucuronopyranoside, medicoside, and quercetin 3-(6‴-*p*-coumaroylglucosyl)(1->2)-rhamnoside 7-glucoside, respectively. 

**Figure 3:**
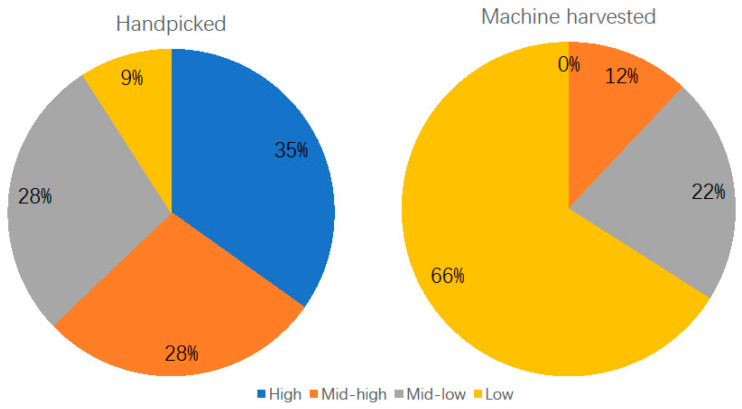
Distribution of sensory grades of oolong tea products made from handpicked or machine-harvested leaves.

To link these metabolite changes to sensory quality, we conducted a sensory evaluation on all samples. The metabolomic differences between handpicked and machine-harvested tea were reflected in the sensory grading. Handpicked groups had a more balanced distribution of all sensory grades with 9% of the finished products being graded as low level, while 66% of the machine-harvested leaves ended as low-grade tea (Figure 3). 

**Figure 4:**
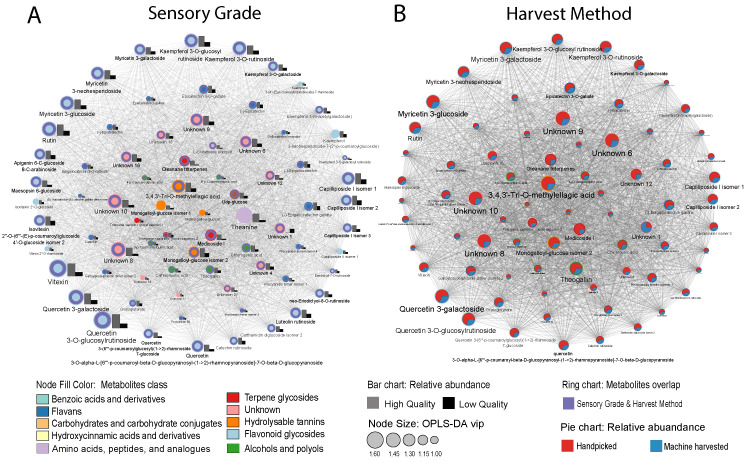
(**A**) Correlation network (|r| > 0.7 and *p* < 0.05) of sensory-related metabolites for high- and low-grade Tieguanyin products based on VIP > 1 metabolites identified in OPLS-DA analysis. (**B**) Correlation network (|r| > 0.7 and *p* < 0.05) of metabolites between handpicked and machine-harvested tea using OPLS-DA. Compound text font is proportional to its VIP value.

The high- and low-grade tea samples were subjected to OPLS-DA analysis. A clear separation was evident between these two groups, as illustrated in Figure 2C. A total of 80 compounds met the criteria for being key discriminant metabolites in differentiating high- and low-grade tea with VIP > 1 and *p* < 0.05. Pearson correlation analysis was performed to construct a metabolites correlation network (|r| > 0.7 and *p* < 0.05; Figure 4A). The top 10 known VIP sensory-related metabolites are quercetin 3-*O*-glucosylrutinoside, vitexin, theanine, quercetin 3-galactoside, rutin, unknown-5, kaempferol 3-*O*-rutinoside, myricetin 3′-glucoside, unknown-7, kaempferol 3-*O*-glucosyl rutinoside, capilliposide I, myricetin 3-neohesperidoside. All of them experienced over 1.5-fold loss between high- and low-grade products. Among them, quercetin 3-*O*-glucosylrutinoside, myricetin 3′-glucoside, quercetin 3-galactoside, rutin, kaempferol 3-*O*-rutinoside, and myricetin 3-neohesperidoside were also the most important (VIP top 10) metabolites for determining the harvesting method (Figure 4B). 

It is not surprising to see that a handpicked approach can produce higher quality tea than the machine-harvesting approach. This has been demonstrated in other high-value crops such as wine [29], olive oil [30], and fruits such as blueberry [31,32], strawberry [33], and table olive [34,35]. Apart from mechanical bruising and a higher green fruit ratio, differences in the percentage of total soluble solids, acidity, and antioxidant levels are also observed between the two groups in these studies [29,30,31,32,33,34,35]. In the wine study, it was found that wine made from handpicked grapes has significantly higher catechin, gallic acid, epicatechin, caftaric acid, and coumaric acid levels than wine made from machine-harvested grapes, especially for catechin, which showed a 50% loss in content [29].

In our study, we found that, for tea, over half of the annotated chemicals showed at least a 1.5-fold change between the handpicked and machine-harvested groups. All the major compounds of tea, such as catechins, theaflavin, gallic acid, chlorogenic acid, and kaempferol-3-glucoside showed at least a 50% reduction. This loss is caused by many reasons. The tea plants, especially Tieguayin, have relatively small and curly leaves [36], which requires a high degree of accuracy and precision for harvesting machines. The production of tea requires younger and more tender shoots [36]; thus, the use of mechanical picking can easily lead to the breakage of new shoots and leaves. This break of plant tissue and cell walls results in chemical leaching and loss during the harvest [37]. Nonselective harvesting of machines will result in the collection of leaves at different mature stages and different parts [37]. Research has shown that the concentration decreases with leaf maturity for gallic acid and caffeine [38]. The traditionally handpicked leaves, which are one bud with two leaves, have the maximum level of amino acids [38]. Tissue-specific metabolic profiles are reported in various studies [19,39]. For example, most kaempferol glycosides are abundant in young leaves but scarce in stems [19]. 

Our data are consistent with the wine research but inconsistent with the previous tea study. The only 1998 study on the impact of mechanization of tea harvesting found only a slight chemical difference between handpicked and machine-harvested tea groups [17]. Hand-plucked teas were slightly richer in biochemical precursors, especially total catechins and polyphenols (less than 1% difference), but had a lower level of total lipid and protein (again less than 1% difference) than shear-plucked tea leaves [17]. Harvesting tea leaves with handheld auto-tools (the shear) can achieve a similar level of selectivity as harvesting by hand. However, such selectivity cannot be achieved when using automatic harvesting machines, such as the Honda Stroke4 used in this study and in the oolong tea industry.

We found that the significant loss of metabolites from the machine-harvested products will result in a lower sensory grade. This is not surprising as the flavor of oolong tea has been reported to be a combination of results of various metabolites. Chemicals such as catechins contribute to the bitterness, while the theaflavins contribute to the briskness. The level of amino acids is related to freshness, and the thearubigin gives the tea a mellow feeling [6]. An overall reduction in secondary metabolite level in tea is likely the cause of the loss in richness in flavor. 

In this study, the most significant metabolites that contribute to this decline in sensory grade are quercetin 3-*O*-glucosylrutinoside, myricetin 3′-glucoside, quercetin 3-galactoside, rutin, kaempferol 3-*O*-rutinoside, and myricetin 3-neohesperidoside. Flavanol glycosides contribute to the bitterness and astringency of tea, especially flavon-3-ol glycosides, which give a velvety and mouth-coating sensation [40]. Common oolong tea processing after collecting leaves involves outdoor withering, indoor withering, pan firing, rolling, drying, and roasting [28,40,41]. Flavan-3-ols are prone to epimerization, oxidative polymerization, and nucleophilic addition reaction during the roasting and fermentation process, which subsequently leads to even lower levels of these chemicals in the final products [41]. 

Considering the overwhelming influence of mechanization on metabolites, proper quality control should be in place to carefully monitor this change during tea production. These six most significant compounds have great potential as marker compounds for monitoring the quality change during tea production when using a targeted chemical analysis approach. Besides proper quality control, field treatments can be applied in the plantations to medicate this loss. In the wine industry, the application of SO_2_ and oenological tannins has been shown to help prevent loss caused by mechanical harvesting. 

### 2.2. Chemometric Approach for Separating Handpicked and Machine-Harvested Tea 

Carefully looking at the PCA plot, we can find that it is possible for the machine-harvest group to achieve a similar metabolomic profile as the handpicked group. Considering the large deviation among the machine-harvested group, it is important to establish a quality control system for monitoring the metabolite changes during manufacture. In addition to targeted chemical monitoring using a list of marker compounds, we would also like to explore the possibility of using untargeted metabolomic data with a chemometric approach for quality control.

Chemometric approaches have become increasingly popular in food quality control. The choice of mathematical models plays an important role in the practicability of the system. DD-SIMCA (data driven soft independent modelling of class analogy) is a one-class model which has a wider range of applications compared to the traditional two-class models, for instance, PLS-DA (partial least squares discriminant analysis). Researchers have realized that SIMCA can provide more reliable results and identify false samples more correctly because most of the false samples are new and very often belong to different classes than the predefined ones in PLS-DA models [42]. DD-SIMCA is a relatively easy statistical modelling tool compared to others and requires significantly less computing power. DD-SIMCA with metabolomic data has been proven effective for quality control in many areas, including water quality [43], gasoline quality [44], medicinal plants [45], and food fraud [46].

Mechanization is the inevitable future of tea harvesting. The average tea-picking speed per person for machine tea picking was 57.14 kg/h per person, while the average tea picking speed per person for manual tea picking was 4.7657.14 kg/h per person. Compared to manual tea picking, mechanical tea picking can improve efficiency by more than 10 times [47]. Labor on commercial tea manufacturers can represent up to 75% of the total field cost when the provision of housing and other social benefits is included [11]. Many countries including China are in the transition period from manual labor to mechanization of tea harvesting. Some production sites will perform a second run of leave selection to remove impurities, debris, and off-spec leaves, which greatly improve the product quality. A suitable quality control method to balance the cost of production and the quality of tea products is more in demand than ever. 

**Figure 5:**
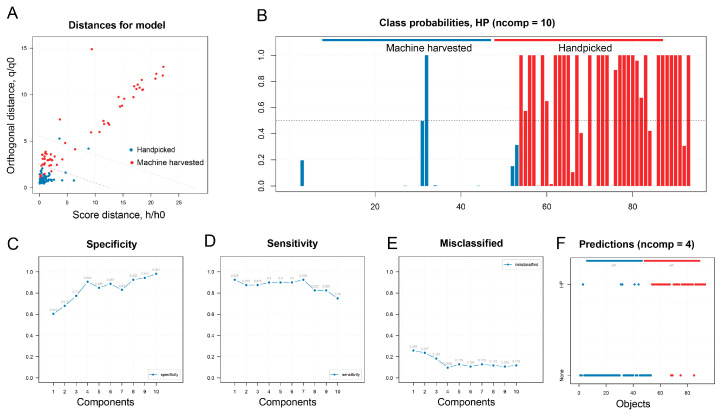
DD-SIMCA models for differentiating oolong tea products made from handpicked or machine-harvested leaves. (**A**) DD-SIMCA score plot; (**B**) class probabilities; (**C**) specificity; (**D**) sensitivity; (**E**) misclassified; (**F**) productions.

Here, we tested the ability of a one-class model (DD-SMICA) to differentiate between handpicked and machine-harvested groups (Figure 5). A total of 92 handpicked Tieguanyin tea products were used as a training set for modelling buildings. The model was calibrated and optimized based on Procrustes cross-validation. We then selected 4 PCs as the optimal number. The accuracy of the training set and validation set was 96.7% and 95.7%, respectively. Later, 40 handpicked teas and 53 machine-harvested samples were used as testing sets. The true positive rate for the handpicked samples was 90.0%, while the true negative rate for the machine-harvested samples was 90.6%. The overall accuracy for the model was 90.3%, indicating a high accuracy for using DD-SIMCA to differentiate between Tieguanyin products made from handpicked or machine-harvested leaves. This study validates the feasibility of using chemometric modelling for tea quality control.

In conclusion, our study provided the necessary scientific base for the movement of harvesting mechanization. The chemical loss caused by current harvesting mechanics is substantial, with at least a 1.5-fold loss of over half of the chemicals. This loss of chemicals will lead to a decline in sensory quality, not to mention the inevitable reduction in bioactivity. Therefore, the industry should take measures to improve the mechanical harvesting tools to mitigate such negative impacts. Quality control measures should be implemented to monitor such changes. Our study identified the six most significant sensory metabolites under the influence of the harvesting method, which can be used as marker compounds for targeted quality control. The untargeted metabolite data combined with DD-SIMCA modelling also provide an alternative approach for general quality monitoring. This knowledge provides solid scientific proof for a commonly accepted industry experience and suggests potential directions for solutions for the tea industry. 

## 3. Materials and Methods

### 3.1. Chemicals and Reagents

Acetonitrile, methanol, and formic acid were purchased from Darmstadt, Germany. Acetic acid was purchased from Tedia Co., Fairfield, OH, USA. A mixture of 18 external standards, including: C ((+)-catechin), GC ((−)-gallocatechin), EC ((−)-epicatechin), EGC ((−)-epigallocatechin), ECG ((−)-epicatechin gallate), EGCG ((−)-epigallocatechin gallate), theaflavin-3,3-digallate, gallic acid, theanine, phenylalanine, chlorogenic acid, caffeic acid, coumaric acid, rutin, trans-ferulic acid, sinapic acid, quercetin, naringenin, and kaempferol, with one internal standard 2′,7′-Dichlorofluorescein were used in this study. The internal standard used in this study was 2′,7′-Dichlorofluorescein. All the standards had a purity level higher than 95%. A detailed list of standards is provided in Table S3. All water used in the study was purified using a Milli-Q AdvantageA10 water purification system (Millipore Sigma, Burlington, MA, USA).

### 3.2. Sample Collection

A total of 185 oolong tea products (*Camellia sinensis* var. *sinensis* cv. ‘Tieguanyin’) were collected from tea production sites in Anxi City, Fujian Province, China. Fresh leaves were harvested by hand or by machine (Honda Sroke 4; Honda Motor Company, Tokyo, Japan) during the same period of 5 days (11 October to 15 October) in the autumn of 2022. The leaf materials were processed into light-scented (Chinese name: Qingxiang) Tieguanyin tea. In the end, a total of 132 tea products were made from handpicked Tieguanyin leaves, while 53 samples were made from machine-harvested Tieguanyin leaves. 

Each sample was created by taking a representative tea product sample, which involved mixing all the leaves collected from that specific sampling batch. After collection, all the tea samples were vacuum-sealed to preserve their freshness and quality. They were then stored in a refrigerator at a temperature of −18 °C (0 °F) before further analysis. 

### 3.3. Sensory Evaluation

The Tieguanyin tea underwent sensory evaluation according to the Chinese standard GB/T 30357.2-2013, utilizing the Gaiwan method [48]. The tea drink was prepared by brewing 5.0 g of Tieguanyin tea with 110 mL of hot water. A team of certified tea evaluators from the Tea Scientific Society of China assessed and scored the quality of all tea products following the same protocol. All tea products were randomly separated into groups of 10 samples. The highest scored tea sample was used as a reference for the next run of evaluation. The certified evaluators first assessed the lid’s fragrance after 1 min of brewing, followed by color and taste evaluation within the next 2 min. Subsequent assessments were conducted after each brewing, with fragrance at 2–3 min, and color and taste at 3 and 5 min post-brewing. The main evaluation considered the second infusion, while the first and third infusions served as secondary references. The sensory scores categorized the samples into four grades: high, mid-high, mid-low, and low (Table S2).

Ethical permission was not required. All participants gave their verbal consent to take part and use their information. All participants have the ability to withdraw from the study at any time. Full disclosure of study requirements and risks was given. 

### 3.4. Sample Preparation

A modified version of the method by Yu et al. (2020) [49] was employed for sample extraction. In brief, 30 mg of finished tea samples were finely ground and lyophilized using a TissueLyser II (QIAGEN Inc., Hilden, Germany). Then the tea samples were extracted with 1 mL of 70% methanol in water. The extraction involved 20 min of sonication at 10 °C, followed by a 10 min centrifugation at the same temperature using a Centrifuge 5430 (Eppendorf Inc., Hamburg, Germany). The resulting supernatant was collected and diluted 50-fold with 70% methanol. This solution was then filtered through a 0.22 µm polyvinylidene fluoride (PVDF) filter (Millipore, Billerica, MA, USA) before LCMS-QToF analysis. All samples were enriched with 250 µL of 200 µg/mL 2′,7′-dichlorofluorescein, which serves as an internal standard. Additionally, a quality control sample was created by pooling equal amounts of all individual samples.

### 3.5. Metabolomics Analysis 

A 1 µL aliquot was injected into a UPLC-QToF-MS system (Waters SYNAPT G2Si; Waters, Milford, MA, USA) with both a Waters photodiode array (PDA) and a SYNAPT G2-Si HDMS QToF mass spectrometer. Metabolites were separated using reverse-phase liquid chromatography on a Waters Acquity UPLC HSS T3 column (100 × 2.1 mm, 1.8 µm). Mobile phase A was 0.1% formic acid with water, and mobile phase B was 0.1% formic acid with acetonitrile. The gradient separation was set as follows: 1–7% B (0–2 min), 7–40% B (2–13 min), 40–60% B (13–17 min), swiftly increased to 99% B at 17 min, maintained at 99% B from 17 to 22 min, and allowed to equilibrate for an additional 3 min before the subsequent injection. Chromatogram solutions from the last 8 min were excluded, and the flow rate was set at 0.3 mL/min. 

Metabolites were ionized via electrospray ionization (ESI) in negative mode, and MassLynxv4.1 software (version 4.1, Waters, Milford, MA, USA) was used for detection. MS data were acquired continuously with collision energy ranging from 10 to 50 eV. Parameters included a capillary voltage of 2.0 kV, cone voltage of 40 eV, collision energy at 4 eV, source temperature of 120 °C, desolvation temperature at 450 °C, cone gas flow of 50 L/h, desolvation gas flow at 800 L/h, and an *m*/*z* range of 50 to 1200 Da. Quality control samples were injected every 10 samples to assess instrument performance.

### 3.6. Data Processing 

The Progenesis QI software (version 2.1; Waters, Milford, MA, USA) was utilized to process raw continuum MS^e^ data, performing tasks such as alignment, picking, normalization, and assignment. Metabolite identification involved a comparison of retention time, accurate masses, MS/MS fragmentation patterns, isotope patterns, and UV absorbance with authentic standards. Online metabolite databases, including Metlin (http://www.metlin.scripps.edu; accessed on 30 November 2023), HMDB (http://www.hmdb.ca/; accessed on 30 November 2023), MassBank (https://massbank.eu/MassBank/; accessed on 30 November 2023), ChemSpider (http://www.chemspider.com/; accessed on 30 November 2023), ReSpect (https://rdrr.io/github/WMBEdmands/compMS2Miner/man/ReSpect.html; accessed on 30 November 2023), and literature references, were also consulted. Detailed annotation information of the metabolites in this study is provided in Table S2. To eliminate the unwanted systematic variations, normalization was performed based on using quality control pool samples (QC). Subsequently, the log10 transformation and auto-scaling (z-transformation), which is mean-centered and divided by the standard deviation of each variable, was applied to normalized intensities. PCA (principal component analysis) and OPLS-DA (orthogonal partial least squares discriminant analysis) analysis were performed in MetaboAnalyst (version 5.0). The Pearson correlation coefficient between metabolites was calculated using R (version 4.3.1) and visualized through Cytoscape (version 3.7.1). Additionally, a DD-SIMCA (Data Driven SIMCA) model was generated based on the R package mdatools (version 0.14.1).

## Data Availability

Data are contained within the article.

## Supplementary Materials

The following supporting information can be downloaded at: https://www.mdpi.com/article/10.3390/plants13040552/s1, Table S1: Annotation information of 182 metabolites in Tieguanyin tea products using UPLC-QToF-MS; Table S2: Four different sensory grades of Tieguanyin tea based on sensory evaluation; Table S3: A full list of standard materials used in UPLC-QToF analysis; Figure S1: Distribution of metabolites classes in Tieguanyin tea products based on untargeted metabolomics using UPLC-QToF-MS.
