# Supplementary material for: The Impact of Harvesting Mechanization on Oolong Tea Quality
Source: Plants (Basel). 2024 Feb 18;13(4):552. doi: 10.3390/plants13040552 (PMC10892732; doi:10.3390/plants13040552)
Supplement: Supplementary file 1 [file plants-13-00552-s001.zip › plants-2834931-SI.pdf]

The impact of harvesting mechanization on oolong tea quality

Junlin Zhou[1], Shuilian Gao[1,2], Zhenghua Du[1], Tongda Xu[1,*], Chao Zheng[1,*],Ying Liu[1,*]

[1,] College of Horticulture, Haixia Institute of Science and Technology, Fujian Agriculture and Forestry University, Fuzhou, 350007, China.

[2], Anxi College of Tea Science, Fujian Agriculture and Forestry University, Fuzhou, 350007, China.

[*,] Correspondence: Tongda Xu (email: tdxu@sibs.ac.cn); Chao Zhen (email: zhengchaotea@fafu.edu.cn) and Ying Liu (email: ying.liu@fafu.edu.cn)

Table S1: Annotation information of 182 metabolites in Tieguanyin tea products using UPLC-QToF-MS

Compound	Putative metabolites	RT (min)	Formula	Theoretical [M - H]- (m/z)	Detected [M - H]- (m/z)	ppm error	MS/MS fragments	Reference
**Benzene and substituted derivatives**								
1	Gallic acid	2.74	$C_7H_6O_5$	169.0137	169.0143	3.71		Authentic standard
2	Theacitrin A isomer 1	4.84	$C_{37}H_{28}O_{18}$	759.1197	759.1205	1.08	343.0458	HMDB
3	Theacitrin A derivatives	4.85	$C_{17}H_{12}O_8$	343.0454	343.0459	1.45		HMDB
4	Methyl gallate	5.38	$C_8H_8O_5$	183.0293	183.0297	1.94		Authentic standard
5	Theacitrin A isomer 2	6.82	$C_{37}H_{28}O_{18}$	759.1197	759.1223	3.41	/125, 177, 289, 423/169, 177, 289, 305, 423	HMDB
**Carboxylic acids and derivatives**								
1	Theanine	1.43	$C_7H_{14}N_2O_3$	173.0926	173.0933	4.16	155.0815	Authentic standard
2	L-Glutathione oxidized	2.19	$C_{20}H_{32}N_6O_{12}S_2$	611.1441	611.1451	1.65		Authentic standard
**Cinnamic acids and derivatives**								
1	3-p-Coumaroyl-1,5-quinolactone	8.32	$C_{16}H_{16}O_7$	319.0818	319.0821	0.91		(Yang, Wang, Cao, Song,

								Xu, & Lin, 2023)
**Flavonoids(Flavans)**								
1	Theasinensin C	2.52	$C_{30}H_{26}O_{14}$	609.1244	609.1255	1.75	471.0932	(Dou, Lee, Tzen, & Lee, 2007)
2	Gallocatechin hexoside	3.43	$C_{21}H_{24}O_{12}$	467.1190	467.1194	0.82		(Martini, Conte, & Tagliazucchi, 2018)
3	Prodelphinidin B isomer 2	3.51	$C_{30}H_{26}O_{14}$	609.1244	609.1256	1.96	441.0812	(Chen, Lin, Liu, Gong, Wang, Li, et al., 2018)
4	(+)-Gallocatechin	3.85	$C_{15}H_{14}O_{7}$	305.0661	305.0666	1.55		Authentic standard
5	Prodelphinidin B isomer 1	4.14	$C_{30}H_{26}O_{14}$	609.1244	609.1252	1.26		(Chen, Lin, et al., 2018)
6	Theasinensin B	4.40	$C_{37}H_{30}O_{18}$	761.1354	761.1358	0.51	591.1127	(Dou, Lee, Tzen, & Lee, 2007)
7	Gallocatechin-3-O-hexoside	4.56	$C_{15}H_{14}O_{7}$	467.1268	467.1192	-16.17	357.0558 (23), 305.0858 (100), 287.0436 (71), 125.0215 (79)	HMDB

8	Procyanidin B1	4.82	$C_{30}H_{26}O_{13}$	577.1346	577.1358	2.10		Authentic standard
9	Epicatechin -gallocatechin dimer isomer 4	4.82	$C_{30}H_{26}O_{12}$	593.1295	593.1305	1.77		(Chen, Lin, et al., 2018)
10	(-)-Epigallocatechin isomer 1	4.94	$C_{15}H_{14}O_{7}$	305.0675	305.0671	-1.27		Authentic standard
11	(-)-Epigallocatechin isomer 2	4.94	$C_{15}H_{14}O_{7}$	611.1401	611.1410	1.52	305.0688 ([M-H]-)	Authentic standard
12	Epicatechin -gallocatechin dimer isomer 5	4.96	$C_{30}H_{26}O_{13}$	593.1295	593.1277	-3.00		(Chen, Lin, et al., 2018)
13	Galloylprodelphinidin dimer isomer 2	4.98	$C_{37}H_{30}O_{18}$	761.1354	761.1357	0.38	609.1237, 591.1138, 423.0714	(Dai, Qi, Yang, Lv, Guo, Zhang, et al., 2015)
14	Procyanidin B3	5.13	$C_{30}H_{26}O_{12}$	577.1346	577.1355	1.52		(Yang, Wang, Cao, Song, Xu, & Lin, 2023)
15	Galloylprodelphinidin dimer isomer 1	5.14	$C_{37}H_{30}O_{18}$	761.1354	761.1358	0.56		(Dai, et al., 2015)
16	Procyanidin C1	5.28	$C_{45}H_{38}O_{18}$	865.1980	865.1988	0.97		HMDB
17	Catechin	5.37	$C_{15}H_{14}O_{6}$	289.0712	289.0716	1.29		Authentic standard
18	Procyanidin trimer isomer 1	5.54	$C_{45}H_{38}O_{18}$	865.2039	865.1990	-5.62		HMDB

19	Theasinensin A isomer 2	5.64	$C_{44}H_{34}O_{22}$	913.1463	913.1469	0.66	169, 177, 305, 423	(Cheng, Yang, Chen, Zhang, Chen, Wang, et al., 2020)
20	Procyanidin	5.69	$C_{30}H_{26}O_{13}$	593.1298	407.0770	-313679.80		HMDB
21	Procyanidin B2	5.70	$C_{30}H_{26}O_{12}$	577.1346	577.1355	1.55	451.1026, 425.0873,	Authentic standard
22	Epigallocatechin-(4β->8)-epicatechin 3-O-gallate	5.86	$C_{37}H_{30}O_{17}$	745.1405	745.1408	0.43	149, 177, 289, 301, 443	HMDB
23	Theasinensin A isomer 1	5.87	$C_{44}H_{34}O_{22}$	913.1463	913.1471	0.86	125, 177, 289, 423	HMDB
24	Procyanidin trimer isomer 3	6.00	$C_{45}H_{38}O_{18}$	865.1980	865.1985	0.55		(Chen, Lin, et al., 2018)
25	(E) Galocatechin-(E) catechin gallate dimer isomer 1	6.06	$C_{37}H_{30}O_{17}$	745.1405	745.1408	0.41		(Chen, Li, Zheng, Wang, Lin, Wang, et al., 2018)
26	(-)-Epicatechin	6.28	$C_{15}H_{14}O_{6}$	289.0712	289.0719	2.42		Authentic standard
27	N-ethyl-2-pyrrolidinone-substituted gallocatechin	6.31	$C_{21}H_{23}NO_{8}$	416.1353	416.1346	-1.74		HMDB
28	Theaflavic acid	6.32	$C_{21}H_{16}O_{10}$	427.0665	427.0664	-0.18		(Yang, Wang, Cao, Song, Xu, & Lin, 2023)

29	(-)-Epigallocatechin gallate	6.36	$C_{22}H_{18}O_{11}$	457.0849	457.0852	0.66	915.1630 ([2M-H]-), 305.0670, 169.0151, 125.0251	Authentic standard
30	3-Galloylprocyanidin B1	6.47	$C_{37}H_{30}O_{16}$	729.1456	729.1460	0.49		HMDB
31	Procyanidin trimer isomer 2	6.52	$C_{45}H_{38}O_{18}$	865.1980	865.1984	0.45		(Chen, Lin, et al., 2018)
32	Oolonghomobisflavan A/ B	6.65	$C_{45}H_{36}O_{22}$	927.1620	927.1622	0.17		HMDB
33	N-ethyl-2-pyrrolidinone-substituted epigallocatechin/gallocatechin isomer 1	6.70	$C_{21}H_{23}NO_{8}$	416.1345	416.1346	0.34		(Yang, Wang, Cao, Song, Xu, & Lin, 2023)
34	8-C-Ascorbylepigallocatechin 3-gallate	6.70	$C_{28}H_{24}O_{17}$	631.0935	631.0957	3.50	479.0598	(Chen, Li, et al., 2018)
35	3-Galloylgallocatechin	6.71	$C_{22}H_{18}O_{11}$	915.1620	915.1604	-1.77		Authentic standard
36	Procyanidin tetramer (B type)	6.73	$C_{60}H_{50}O_{24}$	1153.2614	1153.2628	1.25		(Chen, Lin, et al., 2018; Fraser, Harrison, Lane, Otter, Hemar, Quek, et al., 2012)

37	Epicatechin gallate-epigallocatechin gallate dimer	6.73	$C_{44}H_{34}O_{21}$	897.1514	897.1520	0.71		HMDB
38	N-ethyl-2-pyrrolidinone-substituted epigallocatechin/gallocatechin isomer 2	6.80	$C_{21}H_{23}NO_8$	416.1345	416.1346	0.25		(Yang, Wang, Cao, Song, Xu, & Lin, 2023)
39	Galloylprocyanidin dimer isomer 1	6.80	$C_{37}H_{30}O_{16}$	729.1456	729.1459	0.44		(Chen, Li, et al., 2018)
40	Prodelphinidin A2 3'-gallate	7.03	$C_{37}H_{28}O_{18}$	759.1197	759.1268	9.34	607.1103,589.0945	HMDB
41	(-)-Epiafzelechin	7.24	$C_{15}H_{14}O_5$	273.0763	273.0766	1.22		Authentic standard
42	Fisetinidol-(4α->8)-catechin-3-O-gallate	7.37	$C_{37}H_{30}O_{15}$	713.1506	713.1492	-2.02		(Sawada, Akiyama, Sakata, Kuwahara, Otsuki, Sakurai, et al., 2009)
43	N-ethyl-2-pyrrolidinone-substituted gallocatechin gallate isomer 1	7.40	$C_{28}H_{27}NO_{12}$	568.1455	568.1462	1.27		(Yang, Wang, Cao, Song, Xu, & Lin, 2023)
44	N-ethyl-2-pyrrolidinone-substituted	7.42	$C_{21}H_{23}NO_7$	400.1396	400.1397	0.34		(Yang, Wang, Cao, Song,

	epicatechin/catechin isomer 1							Xu, & Lin, 2023)
45	Procyanidin trimer isomer 4	7.44	$C_{45}H_{38}O_{18}$	865.1980	865.1983	0.34		HMDB
46	Epigallocatechin 3-(3-O-methylgallate)	7.45	$C_{23}H_{20}O_{11}$	471.0927	471.0930	0.58	305.0660, 287.0556	Authentic standard
47	Epiafzelechin 3-O-gallate-(4β->6)-epigallocatechin 3-O-gallate	7.52	$C_{44}H_{34}O_{20}$	881.1565	881.1572	0.79	711.1386	(Chen, Lin, et al., 2018)
48	(E) Galocatechin-(E) catechin gallate dimer isomer 2	7.67	$C_{37}H_{30}O_{17}$	745.1405	745.1409	0.53		(Chen, Li, et al., 2018)
49	N-ethyl-2-pyrrolidinone-substituted epicatechin/catechin isomer 2	7.73	$C_{21}H_{23}NO_7$	400.1396	400.1397	0.20		(Yang, Wang, Cao, Song, Xu, & Lin, 2023)
50	N-ethyl-2-pyrrolidinone-substituted gallocatechin gallate isomer 2	7.75	$C_{28}H_{27}NO_{12}$	568.1455	568.1463	1.42		(Yang, Wang, Cao, Song, Xu, & Lin, 2023)
51	Epicatechin 3-O-gallate	7.87	$C_{22}H_{18}O_{10}$	441.0825	441.0827	0.41	289.0717, 271.0612, 169.0148, 125.0248	Authentic standard
52	(E) Galocatechin-(E) catechin gallate dimer isomer 3	7.87	$C_{37}H_{30}O_{17}$	745.1405	745.1414	1.16		(Chen, Li, et al., 2018)

53	N-ethyl-2-pyrrolidinone-substituted epicatechin/catechin isomer 3	7.88	$C_{21}H_{23}NO_7$	400.1396	400.1396	0.08		(Yang, Wang, Cao, Song, Xu, & Lin, 2023)
54	N-ethyl-2-pyrrolidinone-substituted gallocatechin gallate isomer 3	7.90	$C_{28}H_{27}NO_{12}$	568.1455	568.1463	1.39		(Yang, Wang, Cao, Song, Xu, & Lin, 2023)
55	Theacitrin 3-gallate	8.14	$C_{37}H_{28}O_{17}$	743.1248	743.1253	0.69		(Yassin, Koek, & Kuhnert, 2014)
56	Theasinensin di-gallate	8.17	$C_{44}H_{34}O_{19}$	865.1616	865.1624	0.98		HMDB
57	Epigallocatechin 3-O-caffeate	8.37	$C_{24}H_{20}O_{10}$	467.0978	467.0982	0.87		(Hashimoto, Nonaka, & Nishioka, 1989)
58	Galloylprocyanidin dimer isomer 2	8.90	$C_{37}H_{30}O_{16}$	729.1456	729.1454	-0.30		(Chen, Li, et al., 2018)
59	Epicatechin 3-(3-O-methylgallate)	8.93	$C_{23}H_{20}O_{10}$	455.0978	455.0979	0.29		(Chen, Li, et al., 2018)
60	Epiafzelechin 3-gallate	8.98	$C_{22}H_{18}O_9$	425.0873	425.0874	0.16	273.076	(Chen, Li, et al., 2018)
61	Theaflavin	10.66	$C_{29}H_{24}O_{12}$	564.1268	564.1199	-12.19		HMDB
62	Isoneotheaflavin 3-O-gallate	11.03	$C_{36}H_{28}O_{16}$	715.1299	715.1306	1.03		Authentic standard

63	Theaflavin 3,3'-digallate	11.21	$C_{43}H_{32}O_{20}$	867.1409	867.1417	0.91		Authentic standard
64	neo-theaflavin 3-O-gallate	11.26	$C_{36}H_{28}O_{16}$	715.1299	715.1304	0.65		HMDB
65	(epi) Catechin- (epi) gallocatechin	16.94	$C_{30}H_{26}O_{13}$	593.1295	593.1300	0.76	467.1 (3), 423.07 (100), 355.08 (8), 305.07 (99), 289.07 (9), 261.08 (10), 219.07 (15), 161.02 (16), 137.02 (15), 125.02 (88)	HMDB
**Flavonoids(Flavonoid glycosides)**								
66	Carthamidin diglucoside isomer 1	5.37	$C_{27}H_{32}O_{16}$	611.1612	611.1621	1.41	491.118	HMDB
67	Eriodictyol-7-O-rutinoside	5.75	$C_{27}H_{32}O_{15}$	595.1663	595.1669	1.06	269.0876, 287.0995	HMDB
68	Carthamidin diglucoside isomer 2	6.10	$C_{27}H_{32}O_{16}$	611.1612	611.1621	1.39	491.1195,	HMDB
69	neo-Eriodictyol-8-O-rutinoside	6.18	$C_{27}H_{32}O_{15}$	595.1663	595.1666	0.53	577.1552, 475.1243, 433.1348, 381.0827, 313.0923	HMDB
70	Vitexin isomer 1	6.42	$C_{21}H_{20}O_{10}$	431.0978	431.0979	0.18	283, 311/ 293, 311, 341;	HMDB
71	Maesopsin 6-glucoside	6.58	$C_{21}H_{22}O_{11}$	449.1084	449.1086	0.55		HMDB
72	Vitexin isomer 2	6.60	$C_{21}H_{20}O_{10}$	431.0978	431.0979	0.21	283, 311/ 293, 311, 341;	HMDB

73	Apigenin 6-C-glucoside 8-C-arabinoside	6.95	$C_{26}H_{28}O_{14}$	563.1401	563.1407	1.06		(Chen, Lin, et al., 2018)
74	Myricetin 3-neohesperidoside	6.98	$C_{27}H_{30}O_{17}$	625.1404	625.1411	1.09		(Chen, Li, et al., 2018)
75	Myricetin 3'-glucoside	7.16	$C_{21}H_{20}O_{13}$	479.0826	479.0828	0.41	316.0217	(Chen, Li, et al., 2018)
76	Quercetin 3-(6"-p-hydroxybenzoylgalactoside) isomer 1	7.27	$C_{28}H_{24}O_{14}$	583.1089	583.1097	1.31	463.0876, 300.0274	HMDB
77	Isovitexin 2"-O-glucoside	7.40	$C_{27}H_{30}O_{15}$	593.1506	593.1514	1.26		HMDB
78	Quercetin 3-O-glucosylrutinoside	7.42	$C_{33}H_{40}O_{21}$	771.1984	771.1989	0.67		(Chen, Li, et al., 2018)
79	Quercetin 3-(6"-p-hydroxybenzoylgalactoside) isomer 2	7.64	$C_{28}H_{24}O_{14}$	583.1089	583.1100	1.95	463.0876, 300.0274	HMDB
80	Quercetin 3-O-(6"-galloyl)-β-D-galactopyranoside	7.65	$C_{28}H_{24}O_{16}$	615.0986	615.0993	1.21	463.0879, 313.0565,301.0346, 169.0142	HMDB
81	Chafuroside B	7.66	$C_{21}H_{18}O_{9}$	413.0869	413.0875	1.55	413, 293, 117	(Ishida, Wakimoto, Kitao, Tanaka, Miyase, & Nukaya, 2009)

82	Dihydromyricetin 3-O-rhamnoside	7.67	$C_{21}H_{22}O_{12}$	465.1035	465.1035	0.02	301	HMDB
83	Vitexin 2"-O-rhamnoside	7.74	$C_{27}H_{30}O_{14}$	577.1557	577.1561	0.62		Authentic standard
84	Rutin	7.75	$C_{27}H_{30}O_{16}$	609.1456	609.1457	0.24		Authentic standard
85	Kaempferol 3-O-galactosyl rutinoside	7.78	$C_{33}H_{40}O_{20}$	755.2035	755.2036	0.12	533.1312	(Chen, Li, et al., 2018)
86	Isovitexin	7.81	$C_{21}H_{20}O_{10}$	431.0978	431.0979	0.23		Authentic standard
87	Kaempferol deoxyhexose-hexose-deoxyhexose	8.00	$C_{33}H_{40}O_{19}$	740.2164	740.2088	-10.24		(Sawada, et al., 2009)
88	Catechin rutinoside	8.01	$C_{27}H_{34}O_{15}$	597.1819	597.1828	1.49		HMDB
89	Kaempferol 3-O-glucosyl rutinoside	8.06	$C_{33}H_{40}O_{20}$	755.2035	755.2038	0.41	285.0392	(Chen, Li, et al., 2018)
90	Quercetin 3-galactoside	8.07	$C_{21}H_{20}O_{12}$	463.0955	463.0872	-17.97	109.02977 146.9391 227.03554 243.0289 255.02904 271.02454	HMDB
91	Kaempferol 3-O-rutinoside	8.49	$C_{27}H_{30}O_{15}$	593.1506	593.1513	1.16		Authentic standard
92	Kaempferol 3-O-galactoside	8.57	$C_{21}H_{20}O_{11}$	447.0927	447.0929	0.47		(Chen, Li, et al., 2018)
93	Astragalin gallate	8.59	$C_{28}H_{24}O_{15}$	599.1037	599.1045	1.29		HMDB

94	Kaempferol 3-O-glucoside	8.83	$C_{21}H_{20}O_{11}$	447.0927	447.0928	0.21		Authentic standard
95	Quercetin 3-(6'''-p-coumaroylglucosyl)(1->2)-rhamnoside 7-glucoside isomer 1	9.73	$C_{42}H_{46}O_{23}$	917.2352	917.2357	0.60		(Hasler, Gross, Meier, & Sticher, 1992)
96	Capilliposide I isomer 3	9.80	$C_{48}H_{56}O_{27}$	1063.2931	1063.2942	1.01	917.2332,531.1418, 458.1098, 301.0345 (quercetin)	HMDB
97	Quercetin 3-triglucoside-7-rhamnoside-p-coumaroyl	9.81	$C_{48}H_{56}O_{28}$	1079.2880	1079.2888	0.71	301.0350 (quercetin)	(Martinez-Sanchez, Gil-Izquierdo, Gil, & Ferreres, 2008)
98	Capilliposide I isomer 1	10.03	$C_{48}H_{56}O_{27}$	1063.2931	1063.2936	0.49	917.2364, 531.1427, 301.0354 (quercetin)	(Xie, Xu, Luo, Zhong, & Yang, 2002)
99	Quercetin 3-O-(2G-p-coumaroyl-3G-O-β-L-arabinosyl-3R-O-β-D-glucosylrutinoside)	10.12	$C_{47}H_{54}O_{27}$	1049.2774	1049.2792	1.68		(Mihara, Mitsunaga, Fukui, Nakai, Yamaji, & Shibata, 2004)
100	Capilliposide II isomer 1	10.29	$C_{48}H_{56}O_{26}$	1047.2982	1047.2987	0.48	901.2408 ([M-H-deoxyhexose]-), 523.1451, 431.0963	(Chen, Li, et al., 2018)

101	Quercetin 3-(6'''-p-coumaroylglucosyl)(1->2)-rhamnoside 7-glucoside isomer 2	10.31	$C_{42}H_{46}O_{23}$	917.2352	917.2358	0.70		(Hasler, Gross, Meier, & Sticher, 1992)
102	Kaempferol 3-[2''-(6'''-coumaroylglucosyl)-rhamnoside] 7-glucoside	10.34	$C_{42}H_{46}O_{22}$	901.2402	901.2407	0.60		HMDB
103	Camelliquercetiside A	10.44	$C_{47}H_{54}O_{27}$	1049.2774	1049.2787	1.22		(Manir, Kim, Lee, & Moon, 2012)
104	Camellikaempferoside C isomer 2	10.58	$C_{47}H_{54}O_{26}$	1033.2825	1033.2828	0.30		(Bai, Wang, Wang, Zheng, Wang, Wan, et al., 2017)
105	Kaempferol 3-(4''-(E)-p-coumarylrobinobioside)-7-rhamnoside isomer 1	10.66	$C_{42}H_{46}O_{21}$	885.2453	885.2456	0.34		HMDB
106	Capilliposide I isomer 2	10.68	$C_{48}H_{56}O_{27}$	1063.2931	1063.2939	0.71		(Xie, Xu, Luo, Zhong, & Yang, 2002)
107	2''-(6''-p-Coumaroyl glucosyl) quercitrin	10.72	$C_{36}H_{36}O_{18}$	755.1823	755.1831	1.06		HMDB
108	Kaempferol 3-neohesperidoside-7-(2''-p-coumaroylglucoside)	10.83	$C_{42}H_{46}O_{22}$	901.2402	901.2408	0.70		(Chen, Li, et al., 2018)

109	Camellikaempferoside C isomer 1	10.85	$C_{47}H_{54}O_{26}$	1033.2825	1033.2829	0.41		(Chen, Li, et al., 2018)
110	Capilliposide II isomer 2	10.97	$C_{48}H_{56}O_{26}$	1047.2982	1047.2989	0.65	901.2399 ([M-H-deoxyhexose]-), 523.1456	HMDB
111	Kaempferol neohesperidoside coumarylglucoside	10.99	$C_{42}H_{46}O_{22}$	901.2402	901.2408	0.68		HMDB
112	6''-(4-Hydroxycinnamoyl) astragalin 4'-glucoside	11.01	$C_{36}H_{36}O_{18}$	755.1823	755.1831	1.09		HMDB
113	Quercetin	11.22	$C_{15}H_{10}O_{7}$	301.0348	301.0352	1.41		Authentic standard
114	Kaempferol 3-(4''-(E)-p-coumarylrobinobioside)-7-rhamnoside isomer 2	11.34	$C_{42}H_{46}O_{21}$	885.2425	885.2460	3.91		HMDB
**Organooxygen compounds(Alcohols and polyols)**								
1	Quinic acid	2.93	$C_{7}H_{12}O_{6}$	191.0556	191.0563	3.56		HMDB
2	Theogallin	2.93	$C_{14}H_{16}O_{10}$	343.0665	343.0669	1.27		Authentic standard
3	3-p-Coumaroylquinic acid	5.18	$C_{16}H_{18}O_{8}$	337.0923	337.0928	1.63		(Chen, Lin, et al., 2018)
4	4-p-Coumaroylquinic acid	6.14	$C_{16}H_{18}O_{8}$	337.0923	337.0928	1.39		(Chen, Lin, et al., 2018)
5	5-p-Coumaroylquinic acid	6.43	$C_{16}H_{18}O_{8}$	337.0923	337.0925	0.53		(Chen, Lin, et al., 2018)

**Organooxygen compounds(Carbohydrates and carbohydrate conjugates)**								
6	Sucrose	1.00	$C_{12}H_{22}O_{11}$	341.1084	341.1092	2.45	191.0554, 179.0557	HMDB
7	Norrubrofusarin 6-β-gentiobioside	5.93	$C_{26}H_{30}O_{15}$	581.1506	581.1515	1.59		HMDB
8	Sambacin	7.02	$C_{26}H_{36}O_{12}$	539.2129	539.2136	1.23		HMDB
9	Phenylethyl primeveroside	7.11	$C_{19}H_{28}O_{10}$	415.1604	415.1592	-2.78		(Chen, Li, et al., 2018)
10	Linalool primeveroside isomer 1	11.31	$C_{21}H_{36}O_{10}$	447.2230	447.2230	0.08	493.2264 ([M-H+FA]-)	(Chen, Lin, et al., 2018)
11	Linalool primeveroside isomer 2	11.58	$C_{21}H_{36}O_{10}$	447.2230	447.2231	0.16	493.2278 ([M-H+FA]-)	(Chen, Lin, et al., 2018)
12	Gnaphaliol glucopyranoside	12.09	$C_{19}H_{24}O_9$	395.1350	395.1376	6.60	https://www.mdpi.com/2218-1989/10/10/403	HMDB
**Terpene glycosides**								
1	Triterpene saponins (2)	14.02	$C_{60}H_{92}O_{29}$	1275.5646	1275.5658	0.91		HMDB
2	Sasanquasaponins II	14.27	$C_{69}H_{86}O_{19}$	1217.5685	1217.5601	-6.92	1117 (13), 1037 (100), 875 (13), 651 (7), 555 (4), 481 (3)	HMDB
3	Assamsaponin E isomer 1	15.79	$C_{59}H_{92}O_{26}$	1215.5799	1215.5804	0.43		HMDB
4	Triterpene saponins (unnamed 1)	15.94	$C_{60}H_{94}O_{27}$	1245.5904	1245.5909	0.37	1083, 1065, 951, 915, 753, 709, 611	HMDB

5	Floratheasaponin D	16.01	$C_{60}H_{94}O_{26}$	1229.5955	1229.5961	0.46	1083, 1067, 1049, 789, 611	HMDB
6	Assamsaponin E isomer 2	16.03	$C_{59}H_{92}O_{26}$	1215.5799	1215.5803	0.35		HMDB
7	Triterpene saponins (novel saponin 2)	16.04	$C_{63}H_{92}O_{27}$	1279.5748	1279.5754	0.49	1243 (5), 1191 (4), 1131 (3), 1117 (50), 1099 (100), 937 (15), 713 (4), 585 (19), 481(4)	HMDB
8	Teaseedsaponin A	16.14	$C_{62}H_{96}O_{28}$	1287.6010	1287.6009	-0.11	1201 (2), 1155 (58), 1107 (100), 975 (19), 751 (16), 637 (12), 451 (6)	HMDB
9	Triterpene saponins (5)	16.16	$C_{66}H_{96}O_{29}$	1351.5959	1351.5954	-0.36		HMDB
10	Teaseedsaponin E	16.20	$C_{55}H_{100}O_{32}$	1271.6119	1271.6066	-4.14	1139, 1121, 1007, 989, 845, 827, 669	HMDB
11	Triterpene saponins (4)	16.23	$C_{66}H_{96}O_{28}$	1335.6010	1335.6018	0.60		HMDB
12	Foliatheasaponin III isomer 1	16.24	$C_{61}H_{94}O_{27}$	1257.5904	1257.5907	0.25		HMDB
13	Triterpene saponins (3)	16.26	$C_{65}H_{94}O_{28}$	1321.5853	1321.5859	0.42		HMDB
14	Triterpene saponins (unnamed 4)	16.31	$C_{70}H_{92}O_{24}$	1315.5900	1315.5958	4.40	1153, 1135, 1021, 1003, 859, 683	HMDB

15	Floratheasaponin A	16.41	$C_{60}H_{94}O_{26}$	1229.5955	1229.5961	0.51	1083, 1035, 951, 933, 789, 611	HMDB
16	Triterpene saponins (novel saponin 3)	16.47	$C_{63}H_{92}O_{27}$	1279.5748	1279.5751	0.25	1243 (5), 1191 (4), 1131 (3), 1117 (50), 1099 (100), 937 (15), 713 (4), 585 (19), 481(4)	HMDB
17	Floratheasaponin J	16.48	$C_{62}H_{96}O_{27}$	1271.6061	1271.6066	0.39	1139, 1121, 1007, 989, 845, 827, 669	HMDB
18	Foliatheasaponin I	16.51	$C_{61}H_{94}O_{27}$	1257.5912	1257.5909	-0.23		HMDB
19	Triterpene saponins (unnamed 2)	16.61	$C_{64}H_{94}O_{26}$	1277.5955	1277.5958	0.20		HMDB
20	Triterpene saponins (novel saponin 1)	16.63	$C_{60}H_{96}O_{28}$	1263.5833	1263.5800	-2.61	1161 (6), 1131 (46), 1083 (100), 951 (23), 727 (5), 631 (11), 599 (18), 451 (2) 2	HMDB
21	Triterpene saponins (9)	16.66	$C_{68}H_{98}O_{30}$	1393.6065	1393.5961	-7.45		HMDB
22	Floratheasaponin H	16.73	$C_{62}H_{96}O_{27}$	1271.6061	1271.6062	0.11		(Yang, Wang, Cao, Song, Xu, & Lin, 2023)
23	Triterpene saponins (8)	16.73	$C_{68}H_{98}O_{29}$	1377.6116	1377.6114	-0.15		HMDB

24	Triterpene saponins (6)	16.76	$C_{67}H_{96}O_{29}$	1363.5959	1363.5959	0.02		HMDB
25	Foliatheasaponin III isomer 2	16.77	$C_{61}H_{94}O_{27}$	1257.5904	1257.5909	0.39	89, 191, 205, 247, 119	HMDB
26	Triterpene saponins (7)	16.86	$C_{67}H_{96}O_{29}$	1363.5959	1363.5960	0.11		HMDB
27	Triterpene saponins (1)	16.93	$C_{59}H_{100}O_{33}$	1335.6069	1335.6013	-4.22		HMDB
28	Theasaponin B1	16.97	$C_{65}H_{94}O_{27}$	1305.5904	1305.5907	0.20		HMDB
29	Triterpene saponins (unnamed 3)	16.98	$C_{64}H_{94}O_{26}$	1277.6039	1277.5954	-6.63		(Yang, Wang, Cao, Song, Xu, & Lin, 2023)
30	Assamsaponin J	17.00	$C_{66}H_{96}O_{27}$	1319.6061	1319.6063	0.18		HMDB
**Pyrimidine nucleotides**								
1	Udp-glucose	1.14	$C_{15}H_{24}N_2O_{17}P_2$	564.8000	565.0486	2.00		HMDB
**Tannins**								
1	Monogalloyl-glucose isomer 1	2.46	$C_{13}H_{16}O_{10}$	331.0665	331.0670	1.39		(Chen, Lin, et al., 2018)
2	Monogalloyl-glucose isomer 2	2.68	$C_{13}H_{16}O_{10}$	331.0665	331.0669	1.30		(Chen, Lin, et al., 2018)
3	3,4,3'-Tri-O-methylellagic acid	2.69	$C_{17}H_{12}O_8$	343.0459	343.0460	0.19		HMDB

4	Methyl-galloyl-glucose	3.68	$C_{14}H_{18}O_{10}$	345.0822	345.0823	0.29	225.9254	(Chen, Li, et al., 2018)
5	Digalloylglucose isomer 2	4.46	$C_{20}H_{20}O_{14}$	483.0775	483.0781	1.18		(Chen, Lin, et al., 2018)
6	Digalloylglucose isomer 3	4.79	$C_{20}H_{20}O_{14}$	483.0775	483.0771	-0.73		(Chen, Lin, et al., 2018)
7	Digalloylglucose isomer 1	5.03	$C_{20}H_{20}O_{14}$	483.0775	483.0779	0.86		(Yang, Wang, Cao, Song, Xu, & Lin, 2023)
8	Ethyl digallate	5.57	$C_{16}H_{14}O_{9}$	349.0561	349.0597	10.20		HMDB
9	1,2,6-Tri-O-galloyl-β-D-glucopyranose	6.68	$C_{27}H_{24}O_{18}$	635.0884	635.0892	1.31		HMDB
**Unknown**								
1	Unknown1	1.99	NA	NA	295.1034	NA		
2	Unknown2	3.59	NA	NA	425.0392	NA		
3	Unknown3	3.69	NA	NA	327.0511	NA		
4	Unknown4	4.64	NA	NA	455.0619	NA		
5	Unknown5	5.61	NA	NA	221.8426	NA		
6	Unknown6	6.40	NA	NA	795.1776	NA		
7	Unknown7	7.13	NA	NA	775.1515	NA		
8	Unknown8	7.34	NA	NA	443.0618	NA		
9	Unknown9	7.62	NA	NA	415.0767	NA		
10	Unknown10	8.05	NA	NA	635.2191	NA		

11	Unknown11	8.13	NA	NA	493.0970	NA		
12	Unknown12	8.84	NA	NA	849.1680	NA		
13	Unknown13	9.91	NA	NA	405.0859	NA		
14	Unknown14	9.96	NA	NA	1079.2884	NA		
15	Unknown15	10.89	NA	NA	443.1224	NA		
16	Unknown16	16.32	NA	NA	1147.5325	NA		

Table S2: Four different sensory grades of Tieguanyin tea based on sensory evaluation.

	High	Mid-high	Mid-low	Low
Sensory score range	87.2-82.1	81.5-78.4	78.3-75.7	75.6-66.9
Total sample number	46	43	48	45

Table S3: A full list of standard materials used in UPLC-QToF analysis

Chemical	Purity	Company
Kaempferol	≥98%	BioBioPha Co., Ltd
Theaflavin-3,3-digallate	>98%	Shanghai yuanye Bio-Technology Co., Ltd
Caffeic acid	>98%	Yuanye Biotechnology Inc.
2',7'-Dichlorofluorescein	>97%	Sigma Aldrich
(+)-catechin	>97%	Sigma Aldrich
(-)-gallocatechin	>97%	Sigma Aldrich
(-)-epicatechin	>97%	Sigma Aldrich
(-)-epigallocatechin	>97%	Sigma Aldrich
(-)-epicatechin gallate	>97%	Sigma Aldrich
(-)-epigallocatechin gallate	>97%	Sigma Aldrich
Theanine	>95%	Sigma Aldrich
Rutin	>95%	Sigma Aldrich
Gallic acid	≥98%	Sigma Aldrich
Phenylalanine	≥98%	Sigma Aldrich
Chlorogenic acid	≥98%	Sigma Aldrich
Coumaric acid	≥98%	Sigma Aldrich
Trans-ferulic acid	≥98%	Sigma Aldrich
Sinapic acid	≥98%	Sigma Aldrich
Quercetin	≥98%	Sigma Aldrich
Naringenin	≥98%	Sigma Aldrich

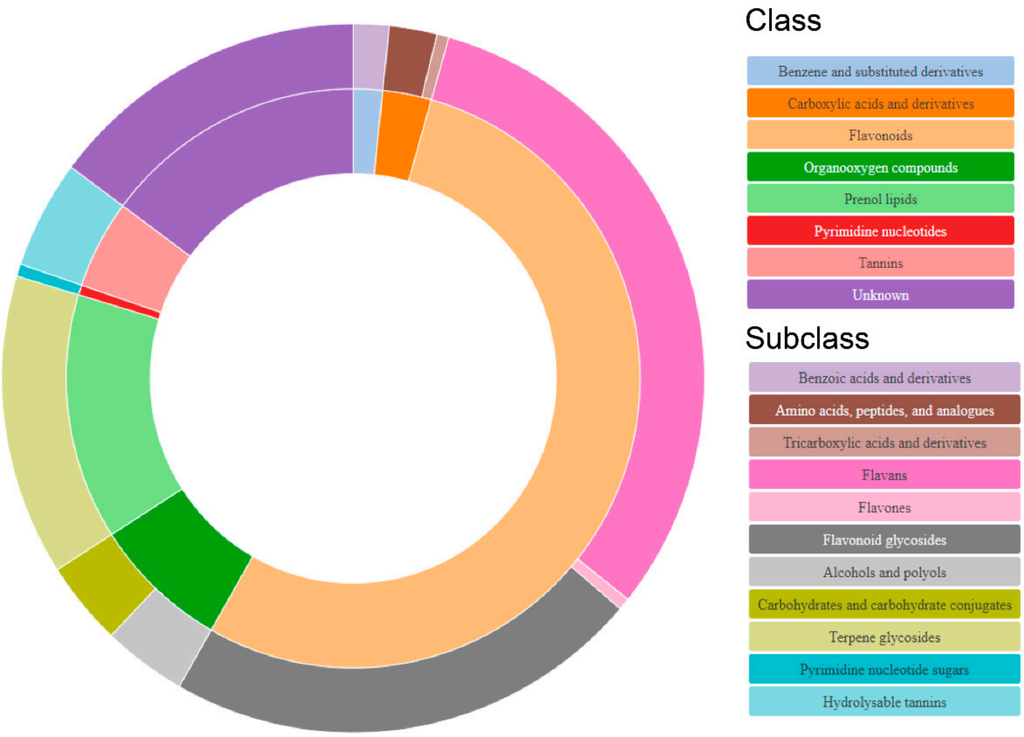

Figure S1: Distribution of metabolites classes in Tieguanyin tea products based on untargeted metabolomics using UPLC-QToF-MS.

Bai, W. X., Wang, C., Wang, Y. J., Zheng, W. J., Wang, W., Wan, X. C., & Bao, G. H. (2017). Novel Acylated Flavonol Tetraglycoside with Inhibitory Effect on Lipid Accumulation in 3T3-L1 Cells from Lu'an GuaPian Tea and Quantification of Flavonoid Glycosides in Six Major Processing Types of Tea. *Journal of Agricultural and Food Chemistry, 65*(14), 2999-3005.

Chen, S., Li, M., Zheng, G., Wang, T., Lin, J., Wang, S., Wang, X., Chao, Q., Cao, S., Yang, Z., & Yu, X. (2018). Metabolite Profiling of 14 Wuyi Rock Tea Cultivars Using UPLC-QTOF MS and UPLC-QqQ MS Combined with Chemometrics. *Molecules, 23*(2), 104.

Chen, S., Lin, J., Liu, H., Gong, Z., Wang, X., Li, M., Aharoni, A., Yang, Z., & Yu, X. (2018). Insights into Tissue-specific Specialized Metabolism in Tieguanyin Tea Cultivar by Untargeted Metabolomics. *Molecules, 23*(7).

Cheng, L., Yang, Q., Chen, Z., Zhang, J., Chen, Q., Wang, Y., Wei, X. J. J. o. A., & Chemistry, F. (2020). Distinct changes of metabolic profile and sensory quality during Qingzhuan tea processing revealed by LC-MS-based metabolomics. *Journal of Agricultural and Food Chemistry, 68*(17), 4955-4965.

Dai, W., Qi, D., Yang, T., Lv, H., Guo, L., Zhang, Y., Zhu, Y., Peng, Q., Xie, D., Tan, J., & Lin, Z. (2015). Nontargeted Analysis Using Ultraperformance Liquid Chromatography-Quadrupole Time-of-Flight Mass Spectrometry Uncovers the Effects of Harvest Season on the Metabolites and Taste Quality of Tea (*Camellia sinensis* L.). *Journal of Agricultural and Food Chemistry, 63*(44), 9869-9878.

Dou, J., Lee, V. S., Tzen, J. T., & Lee, M. R. (2007). Identification and comparison of phenolic compounds in the preparation of oolong tea manufactured by semifermentation and drying processes. *Journal of Agricultural and Food Chemistry, 55*(18), 7462-7468.

Fraser, K., Harrison, S. J., Lane, G. A., Otter, D. E., Hemar, Y., Quek, S.-Y., & Rasmussen, S. (2012). HPLC–MS/MS profiling of proanthocyanidins in teas: A comparative study. *Journal of Food Composition and Analysis, 26*(1-2), 43-51.

Hashimoto, F., Nonaka, G.-i., & Nishioka, I. (1989). Tannins and Related Compounds. LXXVII. : Novel Chalcan-flavan Dimers, Assamicains A, B and C, and a New Flavan-3-ol and Proanthocyanidins from the Fresh Leaves of Camella sinensis L. var. assamica KITAMURA. *Chemical & Pharmaceutical Bulletin, 37*, 77-85.

Hasler, A., Gross, G.-A., Meier, B., & Sticher, O. (1992). Complex flavonol glycosides from the leaves of Ginkgo biloba. *Phytochemistry, 31*(4), 1391-1394.

Ishida, H., Wakimoto, T., Kitao, Y., Tanaka, S., Miyase, T., & Nukaya, H. (2009). Quantitation of chafurosides A and B in tea leaves and isolation of prechafurosides A and B from oolong tea leaves. *Journal of Agricultural and Food Chemistry, 57*(15), 6779-6786.

Manir, M. M., Kim, J. K., Lee, B. G., & Moon, S. S. (2012). Tea catechins and flavonoids from the leaves of *Camellia sinensis* inhibit yeast alcohol dehydrogenase. *Bioorganic & Medicinal Chemistry, 20*(7), 2376-2381.

Martinez-Sanchez, A., Gil-Izquierdo, A., Gil, M. I., & Ferreres, F. (2008). A comparative study of flavonoid compounds, vitamin C, and antioxidant properties of baby leaf Brassicaceae species. *Journal of Agricultural and Food Chemistry, 56*(7), 2330-2340.

Martini, S., Conte, A., & Tagliazucchi, D. J. F. r. i. (2018). Comprehensive evaluation of phenolic profile in dark chocolate and dark chocolate enriched with Sakura green tea leaves or turmeric powder. *FOOD RESEARCH INTERNATIONAL, 112*, 1-16.

Mihara, R., Mitsunaga, T., Fukui, Y., Nakai, M., Yamaji, N., & Shibata, H. (2004). A novel acylated quercetin tetraglycoside from oolong tea (Camelia sinensis) extracts. *Tetrahedron Letters, 45*(26), 5077-5080.

Sawada, Y., Akiyama, K., Sakata, A., Kuwahara, A., Otsuki, H., Sakurai, T., Saito, K., Hirai, M. Y. J. P., & Physiology, C. (2009). Widely targeted metabolomics based on large-scale MS/MS data for elucidating metabolite accumulation patterns in plants. *Plant and Cell Physiology, 50*(1), 37-47.

Xie, C., Xu, L. Z., Luo, X. Z., Zhong, Z., & Yang, S. L. (2002). Flavonol glycosides from Lysimachia capillipes. *Journal of Asian Natural Products Research, 4*(1), 17-23.

Yang, P., Wang, H., Cao, Q., Song, H., Xu, Y., & Lin, Y. (2023). Aroma-active compounds related to Maillard reaction during roasting in Wuyi Rock tea. *Journal of Food Composition and Analysis, 115*, 104954.

Yassin, G. H., Koek, J. H., & Kuhnert, N. J. F. r. i. (2014). Identification of trimeric and tetrameric flavan-3-ol derivatives in the SII black tea thearubigin fraction of black tea using ESI-tandem and MALDI-TOF mass spectrometry. *FOOD RESEARCH INTERNATIONAL, 63*, 317-327.
